# *zmm28* transgenic maize increases both N uptake- and N utilization-efficiencies

**DOI:** 10.1038/s42003-022-03501-x

**Published:** 2022-06-07

**Authors:** Javier A. Fernandez, Jeffrey E. Habben, Jeffrey R. Schussler, Tim Masek, Ben Weers, James Bing, Ignacio A. Ciampitti

**Affiliations:** 1 Department of Agronomy, Kansas State University, Manhattan, KS 66506 USA; 2 Research & Development, Corteva Agriscience, Johnston, IA 50131 USA

**Keywords:** Plant physiology, Field trials

## Abstract

Biotechnology has emerged as a valuable tool in the development of maize (*Zea mays* L.) hybrids with enhanced nitrogen (N) use efficiency. Recent work has described the positive effects of an increased and extended expression of the *zmm28* transcription factor (Event DP202216) on maize yield productivity. In this study, we expand on the previous findings studying maize N uptake and utilization in DP202216 transgenic hybrids compared to wild-type (WT) controls. Isotope ^15^N labeling demonstrates that DP202216 hybrids have an improved N uptake during late-vegetative stages (inducing N storage in lower leaves of the canopy) and, thus, N uptake efficiency (N uptake to applied N ratio) relative to WT. Through both greater N harvest index and reproductive N remobilization, transgenic plants were able to achieve better N utilization efficiency (yield to N uptake ratio). Our findings suggest the DP202216 trait could open new avenues for improving N uptake and utilization efficiencies in maize.

## Abstract (web summary)

Isotope ^15^N labeling demonstrates that *zmm28* transgenic maize have an improved N uptake during late-vegetative stages inducing N storage in lower leaves of the canopy and, thus, N uptake efficiency relative to wild-type maize.

## Introduction

Today, agriculture faces the unprecedented challenge of providing enough safe and nutritious food to nourish an ever-increasing human population in a world threatened by climate change risks^1,2^. The optimization of the nutrient resources to support crop development emerges as a prerequisite for sustaining high productivity while minimizing the environmental footprint of chemical fertilizers^3^. For maize, the largest staple crop in the world^4^, nitrogen (N) has long been recognized as a critical nutrient determining productivity and is expected to do so with the increasing global food demand^5,6^. Therefore, improved N use efficiency (NUE, yield to applied N) is an important and desirable trait for modern hybrids to increase both the economic and environmental sustainability of maize-based cropping systems^7–9^.

Breeding for NUE requires an understanding of physiological traits which can be prioritized as targets for genetic selection, but the genetic-environment (G × E) interactions on these traits bring substantial complexity to this process^10^. Plant NUE can be divided into two physiological mechanisms: (1) the uptake efficiency of N, and (2) the utilization efficiency of N^11^. During uptake, maize roots can acquire N from the soil as nitrate, ammonium, urea, or other organic N forms. The rate of N uptake is related to root architecture and uptake capacity of the plant^12^ and is determined by the N demand of the growing sinks^13^. The most widely adopted indicator of N uptake efficiency in N-fertilized crops is total N absorbed as a proportion of N applied (NUpE). Once incorporated into the plant, N is an essential source for growth as it is used for producing nucleotides, proteins, and numerous cellular components as well as supporting the fixation of carbon dioxide (CO_2_) via photosynthesis. The use of this N accumulated to produce grain yield is commonly evaluated through N utilization efficiency (NUtE, grain yield per unit of N uptake). Research over decades has resulted in substantial progress in understanding the mechanisms underlying both N uptake and N utilization in crops, including the close link between N uptake, assimilation, and remobilization processes^14^. However, the complexity and speed of these reactions at field scale make it difficult to validate promising traits for breeding programs^15^, and NUE improvement promoted by this knowledge is far from being fully exploited^6^. Future progress depends on the ability to elucidate the G × E interactions of candidate genes on N metabolic processes so that they can subsequently be deployed via breeding and/or biotechnology efforts.

The introduction and regulation of N absorption, transport, metabolism, and signaling-related genes by genetic transformation have successfully improved the efficiency of N utilization in plants^16–18^. However, the validation of such early experimental successes in representative field environments will further aid plant biotechnology product development. Recently, the increased and extended expression of *zmm28*, a MADS-box transcription factor, in transgenic *ZmGos2-zmm28* maize improved grain yield across a wide variety of field conditions^19,20^. Z*mm28* is a native MADS-box maize transcription factor, with functions and physiological enhancements described previously in Wu et al.^19^ and Schussler et al.^20^. Since there is no previous publication of a single overexpressed transcription factor increasing NUE in field-grown maize, the extent to which these benefits can be transferred to a variety of environments would represent a major breakthrough for developing more productive and environmentally sustainable crops. In this work, we have investigated the impact of a transgenic maize event (hereafter referred to as DP202216) on N uptake and N utilization in maize crops and their agronomic adaptation to two levels of N supply in the field. These evaluations aim to highlight the connection between N dynamics and productivity in this novel transgenic event and to propose a mechanistic understanding of NUE improvements in maize.

## Results

### DP202216 promotes N uptake and allocation to leaves during vegetative stages

**Fig. 1:**
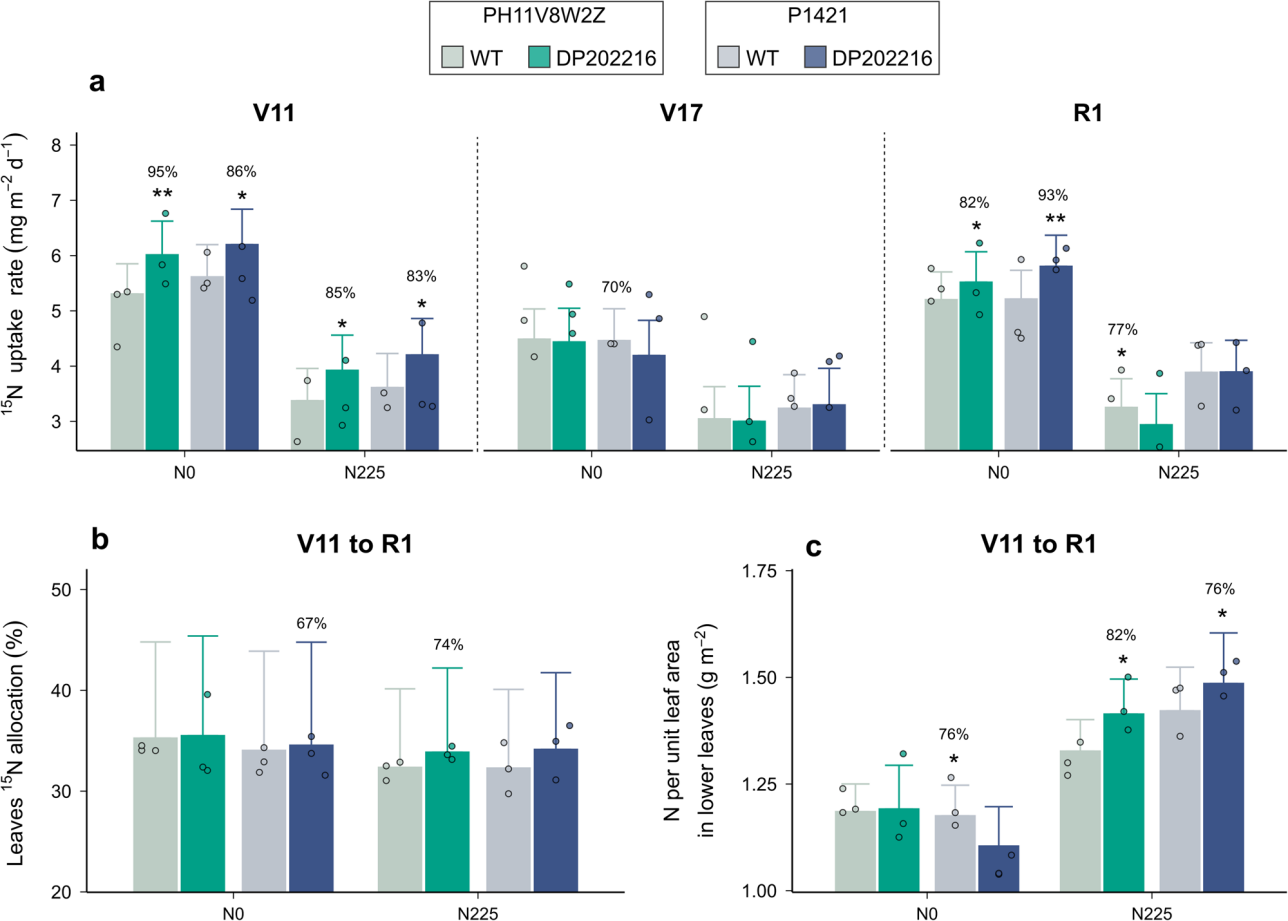
Plant N traits measured from V11 until flowering stage on WT and DP202216 field-grown maize hybrids. **a** Rates of crop ^15^N uptake per day measured using short-term labeling at V11, V17, and R1 on two WT and two DP202216 hybrids under 0 (N0) and 225 (N225) kg N ha^−1^ conditions (*n* = 3 independent samples). **b** Proportion of ^15^N that was allocated to green leaves during the V11-R1 period, expressed in percentage over the total ^15^N uptake (*n* = 3 independent samples). **c** Nitrogen per unit leaf area of the lower section of the canopy during the V11-R1 period (*n* = 3 independent samples). Bars and whiskers represent the medians and standard deviations of the posterior predictive distribution estimated for the two-year data. Asterisks represent moderate (one) or strong (two) evidence for differences between WT and DP202216 maize hybrids.

The seasonal dynamics of N uptake were modeled using short-term ^15^N labeling to determine if DP202216 plays a direct role in N absorption and at which stage the corresponding effects were expressed as affected by N availability. Compared with the wild-type (WT) controls, the most prominent phenotypic trait of DP202216 plants was a higher rate of ^15^N uptake during crop growth stages up to flowering (Fig. 1a). Both at N-unfertilized (N0) and N-fertilized conditions (N225), transgenic plants showed higher rates of ^15^N uptake compared to WT plants with above 83% probability at V11. In contrast, improvements in ^15^N uptake were less consistent during later vegetative stages (V17 until R1). This less pronounced effect of DP202216 as the crop advanced in age correlates with the lack of differences (against the WT controls) in ^15^N uptake rates during the grain filling period (i.e., R3 and R6, Supplementary Fig. 1). In our experimental conditions, a better N uptake capacity of transgenic plants was mainly observed during the rapid vegetative growth of the crop, a phase associated with the formation of ear size for maize. In addition, transgenic plants showed elevated allocation of N to leaves in proportional terms during the V11 to R1 period (Fig. 1b), even though differences with WT controls were small (below 75% probability). These patterns of N allocation combined with the greater N uptake capacity of the transgenic plants suggest DP202216 improves the ability of the crop to accumulate N in leaves during pre-flowering (Supplementary Figs. 2 and 3).

To determine changes in the leaf N profile, the specific leaf N accumulation (SLN, i.e., N per unit leaf area) was characterized at three canopy levels during the same V11 to R1 period. Differences in SLN between transgenic and WT controls were not consistent in the upper and middle sections of the canopy during stages prior to flowering. In contrast, the SLN in the lower leaves of the canopy (i.e., below the ear leaf) was often greater in DP202216 hybrids (Fig. 1c). This improvement occurred especially under N fertilization, where DP202216 hybrids outperformed their WT controls with an 82% (PH11V8W2Z) and 76% (P1421) probability. These results, and the lack of variations in SLN obtained at N-unfertilized, are indicative of a better response to N supply of DP202216 plants on N uptake and storage per unit mass in our trials.

### Ear N demand and reproductive partitioning of DP202216 transgenic maize

**Fig. 2:**
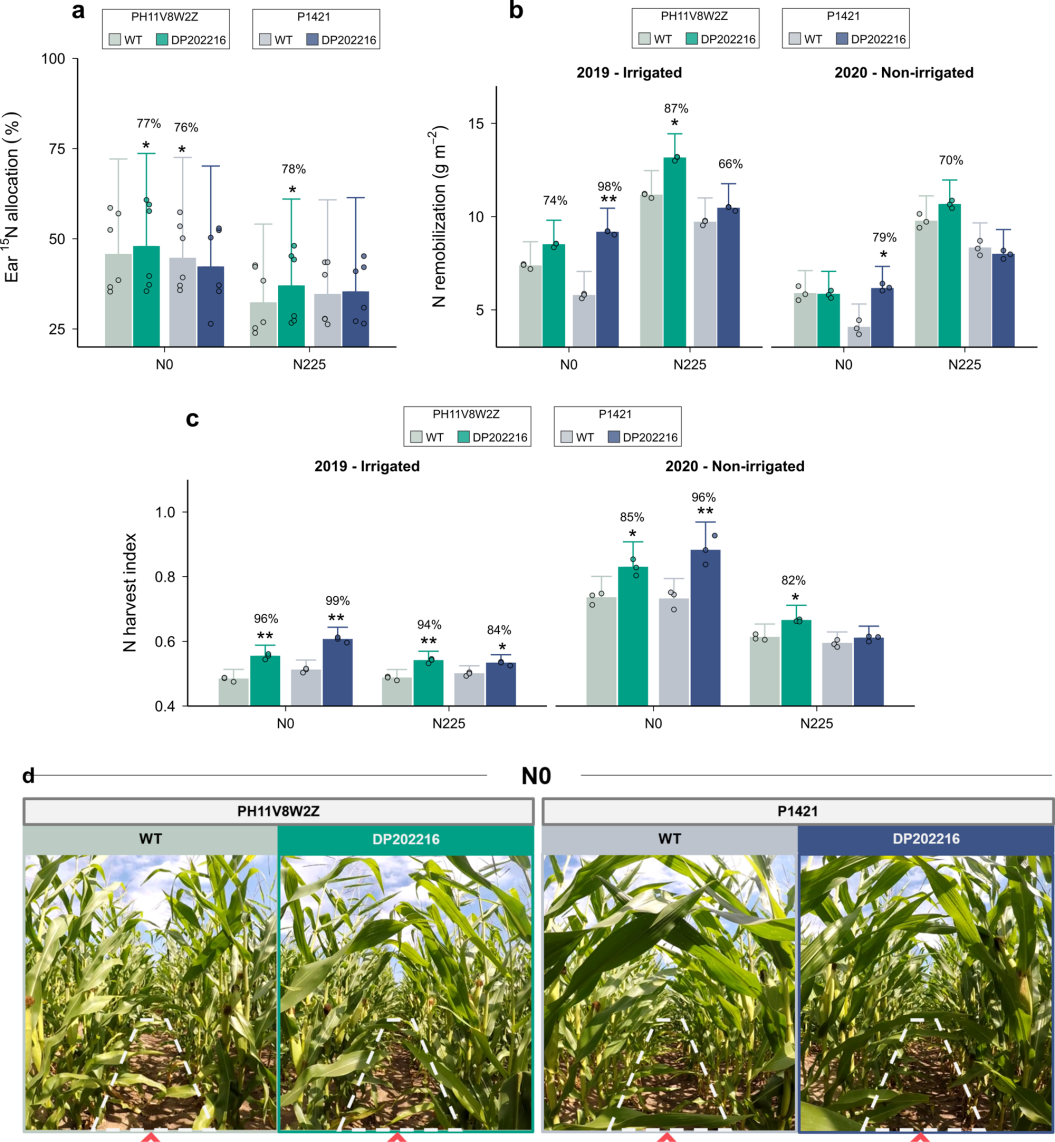
Plant growth and N traits during flowering and post-flowering stages of WT and DP202216 field-grown maize hybrids. **a** Proportion of ^15^N that was allocated to the ear (cob +  grains) during the V17-R6 period, expressed in percentage over the total ^15^N uptake, for two WT and two DP202216 hybrids under 0 (N0) and 225 (N225) kg N ha^−1^ conditions (*n* = 3 independent samples by year). **b** Nitrogen remobilized to the grains from flowering to maturity, calculated using the ‘balance approach’: the difference between vegetative N at flowering (i.e., whole-plant N at flowering) and stover N at maturity (i.e., leaves + stem +  cob + husk N fractions) (*n* = 3 independent samples by year). **c** Nitrogen harvest index, representing the percentage of whole-plant N in the grains at maturity (*n* = 3 independent samples by year). Bars and whiskers represent the medians and standard deviations of the posterior predictive distribution estimated for the two-year data in (**a**), and separated by year in **b** and **c**. Asterisks represent moderate (one) or strong (two) evidence for differences between WT and DP202216 maize hybrids. **d** Image of two WT and two DP202216 hybrids 15 days after flowering under 0 kg N ha^−1^ conditions.

Isotopic labeling during ear and grain development (i.e., from pre-flowering (V17) until physiological maturity (R6)) revealed that transgenic plants had in general a higher N allocation to the ears relative to their WT controls (Fig. 2a). This was the case particularly for PH11V8W2Z genotype, although only moderate evidence for differences was obtained due to the wide range of variation throughout the season in this trait (Supplementary Fig. 2 for the seasonal variation in ^15^N allocation among fractions). It appears DP202216 hybrids achieve the critical period (time around flowering) with an improved source activity (Fig. 2d), which promotes the establishment of sink size potential and triggers an increase in ear N demand during post-anthesis.

Post-flowering N uptake was similar between WT controls and DP202216 plants across most of the experimental conditions evaluated (Supplementary Table 2). The lack of improvements in reproductive N uptake with DP202216 can be explained, at least partially by (1) the high N accumulation observed prior to flowering in shoot tissues (Supplementary Fig. 3), and (2) by the absence of late-season N fertilizer application. Interestingly, under certain conditions (i.e., P1421 under N0), it appears that DP202216 plants were able to reduce N needs without negatively impacting their growth rates and productivity. Instead, transgenic plants were observed to consistently improve N remobilization from the stover to the grains, compared to the WT, with probabilities between 79% and 98% (Fig. 2b). In addition, a site-effect was observed with DP202216 exhibiting a larger improvement in N remobilization under irrigated conditions during 2019 experiment (Supplementary Fig. 4). These findings point toward DP202216 being able to mobilize more N from the stover plant fraction to meet a larger N demand from the grains than their respective WT controls.

Under both N conditions, the DP202216 plants demonstrated a greater N harvest index, i.e., the proportion of whole-plant N accumulated in the grains, relative to their WT controls (Fig. 2c). This response was consistent despite differences in the level of N harvest index (NHI) achieved across 2019 and 2020 experiments (Supplementary Fig. 4). Results showed a large probability for improvement in N harvest index up to 99% (N0) and 96% (N225) probability for the DP202216 hybrids, with a relative increment of 11% on average (i.e., the increment over the N harvest index of WT controls). The DP202216 plants maintained grain N concentrations similar to those of WT plants, 1.26% (N0) and 1.28% (N225) (Supplementary Table 2). These results provide direct evidence of a better partitioning of N to the ear on DP202216 hybrids via enhanced remobilization of assimilates from vegetative tissues to grains, supporting the “homeostasis” of grain N concentration.

### DP202216 increased NUE through improvements in both NUpE and NUtE relative to WT controls

**Fig. 3:**
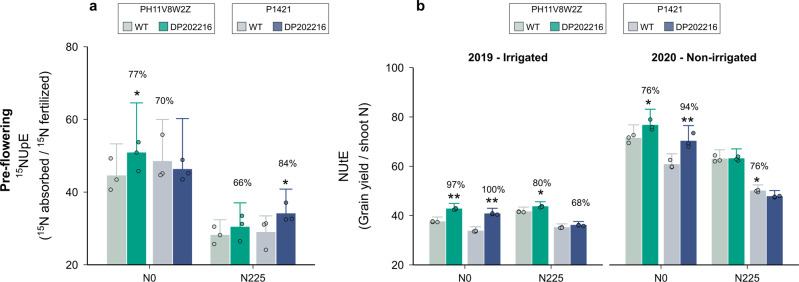
Nitrogen use efficiency of WT and DP202216 field-grown maize hybrids. **a** ^15^N fertilizer uptake efficiency during pre-flowering stages (V11 and V17) expressed in percentage over the total ^15^N applied of two WT and two DP202216 hybrids under 0 (N0) and 225 (N225) kg N ha^−1^ conditions (*n* = 3 independent samples). **b** Nitrogen utilization efficiency calculated as the ratio between grain yield and whole-plant N uptake at maturity (*n* = 3 independent samples). Bars and whiskers represent the medians and standard deviations of the posterior predictive distribution estimated for the two-year data. Asterisks represent moderate (one) or strong (two) evidence for differences between WT and DP202216 maize hybrids.

The fraction of ^15^N fertilizer recovered by the crop (^15^NUpE) was partially enhanced by DP202216 during pre-flowering stages (V11 and V17, Fig. 3a). Evidence for a better ^15^NUpE in transgenic plants was only moderate for PH11V8W2Z under N0 and P1421 under N225. However, the magnitude of the improvement in pre-flowering ^15^NUpE was substantial for these two conditions, with a median increase of 6% in the ^15^N fertilizer recovery. For the remaining hybrid-by-N combinations, pre-flowering ^15^NUpE was similar between transgenic plants compared to WT. During the reproductive period, the capacity of the plant to recover N from fertilizer was similar for both hybrids and both N levels (Supplementary Fig. 1). These outcomes confirm that the capacity to uptake N from the soil during the reproductive period was maintained in the DP202216 event.

A greater NUtE was achieved in transgenic DP202216 plants compared to their WT controls. NUtE improvements in DP202216 plants were especially consistent under N0, outperforming WT controls with above 94% probability across experiments (Fig. 3b). The increases in NUtE with DP202216 were less evident under N225. When crops were N-fertilized, there was a tendency of improvement in NUtE in transgenic plants although with weak or moderate evidence for significance (<90% probability). On average, the effect of DP202216 was more markedly for the 2019 field experiment when lower levels of NUtE were realized (medians of posterior distribution: 39 vs. 63 kg kg^−1^ for 2019 and 2020, respectively). In addition, the larger response in NUtE to N rates in 2020 trials might be attributed to a greater N immobilization and lower soil N mineralization rates derived from wheat residues, although no substantial differences in the soil inorganic N pool were found at planting (Supplementary Table 1). However, taken all together, results across our field trials showed the DP202216 trait increased NUtE in maize crops relative to the WT plants, but more consistently under N limited conditions.

## Discussion

**Fig. 4:**
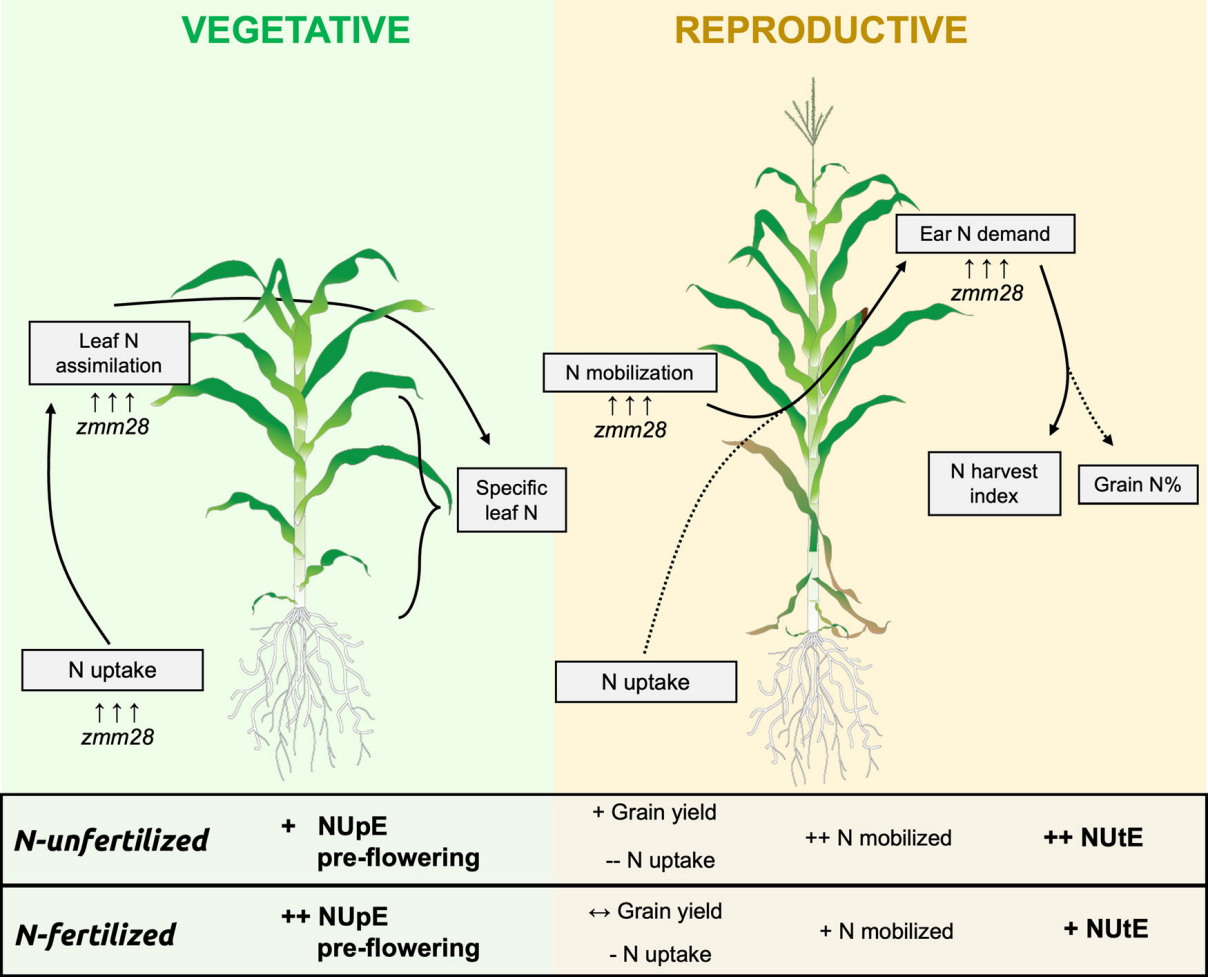
Physiological model of DP202216 effects on NUE during pre- and post-anthesis. Graphical representation describing the critical impacts of DP202216 (*ZmGos2-zmm28*) in NUE during pre- and post-anthesis under N-fertilized and N-unfertilized conditions, compared to WT. Plus (+) symbols indicate increases, minus (−) decreases, and left-right arrows (↔) no changes observed for one or two hybrids (one/two symbols).

In crop plants, N use efficiency involves a complex network of regulatory genes driving N-related processes. Research on the modulation of nitrate uptake and signaling in crops began in the last several years, and MADS-box transcription factors are of interest due to their association with the physiological processes of plants^21–23^. Nonetheless, improvements of NUE in field crops promoted by the genetic manipulation of MADS-box genes have not been exploited. Recently, overexpression of the *zmm28* gene, a MADS-box transcription factor in maize, has been documented to increase nitrogen assimilation by around 17% at the V8 stage in plants growing hydroponically in growth chambers^19^. Here, using field studies, we demonstrate that the altered expression of *zmm28* is an effective strategy to enhance NUE of field-grown maize. DP202216 plants uniquely activate the native *zmm28* gene much earlier (V2) than in the WT (V6), via control of a moderately constitutive GOS2 promoter^19^. We hypothesize that this earlier and increased expression of *zmm28* triggers an early and enhanced uptake of N in the transgenic plants relative to WT, in part due to a differential source N demand. Evidence from this study reveals the DP202216 physiological response of altering the early vegetative N acquisition more efficiently converts N assimilates into grain yield (Fig. 4).

Members of the MADS-box gene family are known to be involved in vegetative growth and development in plants^24–26^. In maize, increased and extended expression of the *ZmSOC1* MADS-box gene was responsible for increased vegetative growth and plant height at flowering^27^. These results are consistent with those of Wu et al.^19^ showing that the upregulation of *zmm28* enhanced plant vigor and leaf area prior to flowering in greenhouse-grown plants. Our multi-year replicated field trial data demonstrate that vegetative growth of DP202216 transgenic hybrids is supported by a stronger acquisition of N from the soil during the rapid stem elongation stage. This corresponds with the *zmm28* protein levels in roots achieving their highest levels around V9 in transgenic plants compared to WT controls^19^. Together, these results establish that the *zmm28* gene product plays an important role in N uptake by varying N supply, a function related to those described for other MADS-box transcription factors mainly through nitrate signaling and lateral root elongation in plants^28–31^. These results, however, rely on the assumption of no interaction year-by-treatment for the V11 and V17 stages in our experiments. It was in order to capture a larger period of the ear and tassel formation during the vegetative phase that we chose to apply ^15^N fertilizer in the second year of experimental trials. This phase corresponds to the greatest expression of the *zmm28* transcript and protein levels beginning at V11^19^. Furthermore, the absence of experimental year- and year-by-treatment effects across the remaining post-flowering stages when ^15^N fertilizer was applied (R1 and R3) is proof that such an assumption tended to be satisfied in our experiments. Nevertheless, as a whole, both experimental sites concur that the altered and extended expression of *zmm28* appears to confer major improvements in N uptake and storage during late-vegetative stages. More importantly, the physiological characterization of DP202216 represents a sustainable approach for recovering N from fertilizers in maize crops, especially for N applied during early vegetative stages.

Increases in vegetative N assimilation resulted in a higher specific N content in the lower leaves of the canopy (above the threshold value for photosynthesis^32^), which suggests an ‘extra N’ pool presumably available for post-anthesis utilization. Likewise, Reyes Ponce^33^ showed that modern hybrids accumulate more N per unit leaf area, especially in leaves below the ear node, compared to older genotypes of the US Corn Belt. While it seems that this strategy has been a major physiological basis of genetic improvement over decades, our study demonstrates that the DP202216 trait may present novel genetic variation for improvement for this N storage and retrieval strategy in maize. This improved N status before flowering not only increases the amount of N available for translocation but also supports the formation of ear size and kernel set^34^. It is therefore expected that, especially under suboptimal N conditions, DP202216 can enhance the establishment of reproductive sink size, compared to WT controls. This is consistent with results from previous studies demonstrating the improved productivity of *ZmGos2-zmm28* transgenic hybrids over multiple limited-N environments^20^.

The ability of plants to absorb and store any excess of mineral N during vegetative growth and then translocate it to the grains has been previously identified as a good marker of productivity in maize^35,36^. This is because grain filling is largely under the control of the N status achieved throughout the early phases of development^37^. In our study, DP202216 transgenic hybrids relied more on the pool of N stored before flowering to fill the kernels compared to WT controls, under the conditions tested. Under the tested conditions, the high protein levels of *zmm28* in leaves but not in roots during reproductive stages reported previously for DP202216 plants^19^ are consistent with these results of elevated N remobilization from the stover during post-flowering (rather than N uptake) in transgenic plants. Altogether, these results suggest that altered *zmm28* expression plays a major role in N assimilation and translocation processes (Fig. 4). While these findings were calculated using the “balance approach”, the complexity of N fluxes within the plant deserves future consideration to differentiate whether changes in the net N remobilization from stover to grains with *zmm28* are more associated with pre-flowering N (accumulated in vegetative tissues) or post-flowering N (allocated first to stover and later mobilized to the grains). Moreover, the distribution of ^15^N during the post-flowering phase and the N harvest index at maturity showed a strong preferential allocation of N to the ear in DP202216 hybrids, relative to their WT controls. This strategy allowed DP202216 hybrids to achieve a similar N concentration in grains at maturity, compared to their WT controls. This DP022216 maintenance of grain N should provide support for more effective use of plant N to produce grain in breeding programs targeting NUtE^37–39^.

Nitrogen use efficiency has been a challenging, and often overlooked, target breeding trait in many crops because of its complexity and large interaction with soil and environmental factors^9^. This trait has been poorly exploited to date but, at the same time, retains a high potential for future improvement. DP202216 transgenic plants have greater yields under sub-optimal N conditions and, perhaps even more importantly, may lower fertilizer requirements to optimize productivity at high N. These findings suggest that DP202216 hybrids could provide a path toward more sustainable maize production worldwide. Based on our analysis, DP202216 (1) augments pre-flowering N assimilation (better N uptake efficiency), and (2) improves post-flowering N use (better N utilization efficiency) (Fig. 4). This, however, is contrary to the historical trend of improvement in NUE mainly through a better efficiency of post-flowering N uptake^38–41^. The simultaneous improvement in uptake and utilization of vegetative N with DP202216 appears, therefore, as a distinctive mechanism to enhance yield potential and yield stability in maize^20^. Recently, Ciampitti and Lemaire^42^ reviewed the importance of focusing on improving the intrinsic N uptake capacity (N uptake per unit of biomass) for maize, targeting as one of the future research priorities further crop breeding improvements on root traits for more effective use of N. In line with this concept, modulating the expression of *zmm28* opens a new potential avenue for maize breeding programs around the world to increase the effective use of N under varying environmental conditions.

## Methods

### Field trials description and experimental design

The field experiments were conducted during the 2019 and 2020 growing seasons at the Corteva Agriscience research station in York NE, USA (40˚53′ N, 97˚35′ W). Soils consist of a silty clay loam (smectitic, mesic Udic Argiustolls) and analyses were conducted at pre-planting to characterize initial conditions (Supplementary Table 1).

Standard agronomic practices were used during the season to optimize yield in each environment. Experiments were carried out under irrigated (2019) and non-irrigated (2020) conditions and the previous crops were soybean (2019) and wheat (2020). In 2019, irrigation was applied with a linear-move sprinkler system based on crop requirements to eliminate any significant plant water deficits. Seeds were planted on 14 May (2019) and 1 May (2020) at a target density of 82,000 (2019) and 70,000 (2020) plants ha^−1^ with a row spacing of 0.76 m. Chemical control was carried out to keep field trials free of weeds, pests, and diseases.

Hybrids PH11V8W2Z and P1421 were selected to represent a sample of two elite Corteva Agriscience maize hybrids having similar relative maturity (114 days). Transformation and backcrossing procedures used to produce the DP202216 transgenic event and wild type (WT) hybrids are described in Wu et al.^19^. Two N rates 0 and 225 kg ha^−1^ (N0 and N225) were applied two days after planting using 28% urea-ammonium nitrate (UAN) liquid N fertilizer as the source. Treatments were arranged in a split-plot design with three repetitions, with N supply as whole-plot and hybrids as subplot. Each experimental unit was an eight-row plot with a size of 5 m long and 6 m wide.

### Multi-stage ^15^N isotopic labeling

Short-term isotope ^15^N labeling was used to determine N uptake and allocation following the methodology employed in de Oliveira Silva et al.^43^ and Fernandez et al.^44^. In 2019, labeling was performed at three key developmental stages:^45^ 10 days before flowering (V17), flowering (R1), and mid-grain-filling (milk stage, R3). In 2020, an additional earlier vegetative measurement was performed at the 11th expanded leaf (V11) stage in addition to samplings at R1, R3, and physiological maturity (R6). Briefly, at each stage, labeled fertilizer Ca(NO_3_)_2_ (10.15% ^15^N) was applied to the soil at the base of a five-plant microplot in each experimental unit at 0.7 g plant^−1^ using plastic syringes. Five days after labeling, the three center plants of each microplot were cut at the ground level and separated into leaves (green leaf blades), stem (stem, leaf sheaths, tassel, and husks), and ears (grains + cob, when present). The period between labeling and sampling was extended to 15 days for the sampling at R6, to account for the lower rates of N absorption at this stage and to ensure that the harvest was performed at physiological maturity in all entries. For all data reported, sampling stages for the labeling procedure refer to stages at which the biomass collection was performed. Samples were dried at 65 ˚C until constant weight and prepared for isotopic analyses.

### Harvesting

Specific leaf N (SLN, i.e., leaf N per unit leaf area) in the canopy profile was determined at each sampling time (in 2019, V17, R1, and R3; in 2020, V11, R1, R3, R6) by sampling twelve leaf disks of 15.89 mm diameter in two (only at V11) or three canopy levels. At V11, the upper section included the three uppermost-expanded leaves while the lower section represented the remainder of plant green leaves. For samplings when the ear was visible, leaves were classified as from the middle section of the canopy (leaf blades from the ear node 0 and ±1 node positions), lower section (leaves from the −2 node and below), and upper (leaves from the +2 node and above). Samples were dried at 65 ˚C until constant weight, weighed on a 0.01 g precision scale, and prepared for N analyses.

At harvest maturity, grain dry matter was collected for N use efficiency parameters on the four center rows that remained unaltered during the season. Grains were then dried at 65 ˚C until constant weight and prepared for laboratory N analyses.

### Laboratory analyses and calculations

All grain and leaf disk materials were ground through a 0.25-mm sieve, and N concentration was determined by combustion on an automated elemental analyzer (FlashEA 1112 N/Protein analyzer, Thermo) in two technical replicates. N concentration was multiplied by the dry weight of the fraction to obtain N content in grains and leaf disks. SLN was obtained as the ratio between leaf disk N content and its known area (1.98 cm^−2^).

For isotopic analysis, tissue samples were ground through a 0.10 mm sieve. Ground samples were weighed (3 mg) and packed in tin capsules with an A&D microbalance (BM-22) with an error index of 0.001 mg. Capsules were introduced into an automated elemental analyzer (PyroCube – Elementar Americas) coupled to an isotope ratio mass spectrometer (visION, Elementar Americas, Ronkonkoma, NY, US). Nitrogen concentration of tissue was obtained along with the isotopic composition of δ^15^N. Percent N values were multiplied by the dry biomass of each fraction (i.e., leaves, stem, and ears) to calculate tissue N content (${{{\mbox{N}}}\,{{\mbox{content}}}}_{{{\mbox{fraction}}}}$). Nitrogen use efficiency indicators and related parameters were calculated using the obtained biomass and N content data (Supplementary Note 1). The ^15^N uptake rates and ^15^${{{\mbox{N}}}\,{{\mbox{allocation}}}}_{{{{{{\rm{fraction}}}}}}}$ were calculated from the differences in ^15^N abundances of samples from labeled plants and non-labeled control plants as in Fernandez et al.^44^. Here, values that represent the ^15^${{{\mbox{N}}}\,{{\mbox{allocation}}}}_{{{\mbox{fraction}}}}$ from a developmental period represent the pooled average across stages included (e.g., for the V11 to R1 period: V11, V17, and R1 stages). For leaf tissue, reported data represents ^15^N allocation across all three-canopy levels (low, middle, and upper nodes).

### Statistics and reproducibility

Bayesian ‘mixed-effects’ models were fitted to the data to quantify the likelihood for differences between treatments in the experiment. Detailed information on the model adjustments, choice of priors, and Markov chain Monte Carlo settings are provided in the Supplementary Information (Supplementary Note 2). Briefly, Bayesian ‘mixed effects’ models were fitted to the data with 'population-level' effects (i.e., 'fixed' effects in a frequentist vocabulary) for N treatment, hybrids, gene expression trait, and growth stage, and with 'group-level' effects (i.e., 'random' effects in a frequentist vocabulary) for Year (*n* = 2 independent years) and Block (*n* = 3 independent replicates) to recognize the experimental structure of the data and imbalances in the sampled growth stages across years. Inferences were based on four chains of the MCMC algorithm, with 4000 iterations and a warmup period of 2000 draws for the MCMC calibration on each. Sampling convergence of the chains was assessed using trace plots and the Gelman-Rubin diagnostics^46^. Medians of the posterior distributions were reported due to their robustness properties. The Bayesian modeling approach allowed determining the expected value of the predicted dynamics with a probabilistic component by means of their posterior distribution of samples^47^. An advantage of this approach is that it does not require a fixed measure of significance (as a *p*-value in a frequentist approach) but instead can provide an exact quantification of the likelihood for differences between treatments^47^. Therefore, the probabilities for differences between DP202216 and WT plants were estimated based on the posterior distribution of pairwise differences [derived from the samples produced by the Markov chain Monte Carlo (MCMC) algorithm] at each condition. Probabilities for differences were reported in figures when above 65% (weak evidence), 75% (moderate evidence), and 90% (strong evidence). In addition, the group-level parameters for the varying-intercepts for year and varying-slopes for year by N treatments, hybrids, and transgenic events were examined using their 90% credible intervals to determine whether each variable varied significantly across years (Supplementary Fig. 4).

### Reporting summary

Further information on research design is available in the Nature Research Reporting Summary linked to this article.

## Supplementary information

Supplementary information

Reporting Summary

## Data Availability

The data that support the findings of this study may be subject to third party ownership and/or regulations from Corteva Agriscience. Data are available from the authors upon reasonable request. Source data and code of the main figures are publicly available in an Open Science Framework (OSF) repository at 10.17605/OSF.IO/WT4SM.
