# Supplementary material for: zmm28 transgenic maize increases both N uptake- and N utilization-efficiencies
Source: Commun Biol. 2022 Jun 7;5:555. doi: 10.1038/s42003-022-03501-x (PMC9174173; doi:10.1038/s42003-022-03501-x)
Supplement: Supplementary file 1 — Supplementary information [file 42003_2022_3501_MOESM1_ESM.pdf]

Supplementary information

Article Title: *zmm28* transgenic maize increases both N uptake- and N utilization-efficiencies

Authors and Affiliations:

Javier A. Fernandez[1*], Jeffrey E. Habben[2], Jeffrey R. Schussler[2], Tim Masek[2], Ben Weers[2], James Bing[2], and Ignacio A. Ciampitti[1*]

Department of Agronomy, Kansas State University, Manhattan, KS 66506 [1] and Research & Development, Corteva Agriscience, Johnston, IA 50131 [2]

Supplementary Note 1

Calculations of ^{15}N abundance and NUE indicators

For the enriched samples, ^{15}N abundance was expressed in atom percent by the ratio:

$$\text{At\%}\left(^{15}\text{N}\right)= \frac{^{15}\text{N}}{^{15}\text{N}+^{14}\text{N}}, \tag{1}$$

and the atom percent excess [At% $\left(^{15}\text{N}\right)$Excess] was calculated as the difference between ^{15}N abundances of samples from labeled plants and non-labeled control plants:

$$\text{At\%}\left(^{15}\text{N}\right)\text{Excess} = \text{At\%}\left(^{15}\text{N}\right)_{\text{labeled sample}} - \text{At\%}\left(^{15}\text{N}\right)_{\text{non-labeled control}} . \tag{2}$$

Total ^{15}N assimilated from labeling was expressed in g m^{-2} and estimated for each tissue as:

$$^{15}\text{N uptake}_{\text{ fraction}} = \text{N content}_{\text{ fraction}} \times \left(\frac{\text{At\%}\left(^{15}\text{N}\right)\text{Excess}_{\text{ fraction}}}{100}\right) . \tag{3}$$

In addition, the relative allocation of ^{15}N to each fraction was expressed in percentage and obtained as:

$$^{15}N\ allocation_{fraction} = \frac{^{15}N\ uptake_{fraction}}{^{15}N\ uptake_{total}} \times 100, \qquad (4)$$

where $^{15}N\ uptake_{total} = \sum {}^{15}N\ uptake_{fraction\ =\ stem,\ leaves,\ ear}$.

Nitrogen use efficiency indicators and related parameters were calculated using the obtained biomass and N content data. Nitrogen remobilization to the grains from flowering to maturity was calculated using the 'balance approach' as the difference between vegetative N at flowering (i.e., whole-plant N at flowering) and stover N at maturity (i.e. leaves + stem + husk + cob N fractions). Cob N content at maturity was calculated as the difference between the ear and grain N content. In addition, N harvest index was calculated as the ratio between whole-plant N and grain N content at maturity expressed in percentage. N utilization efficiency (NutE) was calculated as the ratio between dry matter grain yield and whole-plant N uptake at maturity. N uptake efficiency of ^{15}N fertilizer ($^{15}NUpE$) was calculated as the percentage of ^{15}N absorbed by the plant over the total fertilizer applied at each stage. Pre-flowering $^{15}NUpE$ represents the pooled average of $^{15}NUpE$ across V11 and V17 sampling stages, and post-flowering $^{15}NUpE$ represents the same across R1, R3, and R6 stages.

Supplementary Note 2

Bayesian Modelling

All the data collected were analyzed using R program (version 3.6.1) in RStudio interface [1]. A Generalized Extreme Studentized Deviation (GESD) procedure was conducted on the data to identify and eliminate outliers before statistical analyses [2]. Bayesian 'mixed effects' models were fitted to the data using Stan programming language via *brms* package [3,4]. A Bayesian approach with Markov Chain Monte Carlo (MCMC) sampling was utilized in a model with 'population-level' effects for N treatment, hybrids, gene expression trait, and growth stage (i.e. 'fixed' effects in a

frequentist vocabulary), and 'group-level' effects for Year and Block to recognize the experimental structure of the data (i.e. 'random' effects in a frequentist vocabulary).

Adjustments on the distributional assumption of the variables were taken into consideration for model fitting. For positive continuous quantities taking positive or negative values, process models were fitted with a normal distribution as:

$$[\boldsymbol{y}|\boldsymbol{n}, \boldsymbol{V}] \sim N(\boldsymbol{\mu} = \boldsymbol{n}, \boldsymbol{V}) \,, \qquad (5)$$

where $\boldsymbol{y}$ is the vector of measured observations, $\boldsymbol{\mu}$ is the vector of expected values for the variable at each population-level effect, which is linked to the linear predictor $\boldsymbol{n}$ in an identity function, and $\boldsymbol{V}$ is the variance-covariance matrix. For ^{15}N allocation proportions [i.e. continuous variables between 0 and 1, and for which the sum of all (leaves + stem + ear) is equal to the unity], a Bayesian generalized linear model with a Dirichlet distribution was used for the process model [5]:

$$[\boldsymbol{y_c}|\boldsymbol{n_c}, \boldsymbol{\varphi}] \sim Dirichlet\left(\boldsymbol{\mu_c} = \frac{\exp\,(\boldsymbol{n}_c)}{\sum_{d=1}^{C} \exp\,(\boldsymbol{n}_d)}\,, \boldsymbol{\varphi}\right), \qquad (6)$$

where $\boldsymbol{y_c}$ is a vector of ^{15}N allocation for the cth plant fraction at each population-level combination, $\boldsymbol{\varphi}$ is a vector of positive precision parameters, and $\boldsymbol{\mu_c}$ is the vector of expected ^{15}N allocation linked to the linear predictor $\boldsymbol{n}$ in a multinomial logit function [4]. The expected value for the relative ^{15}N allocation $\boldsymbol{\mu_c}$ is, therefore, between 0 and 1 and subject to the constraint $\sum_{d=1}^{C} \boldsymbol{\mu_d} = 1$ [5].

For all models, the linear predictor $\boldsymbol{n}$ was defined as:

$$\boldsymbol{n} = \boldsymbol{X}\boldsymbol{\beta_f} + \boldsymbol{Z}\boldsymbol{\beta_r}$$

where $\boldsymbol{X}$ and $\boldsymbol{Z}$ are design matrices of covariates and random-effects in the model, $\boldsymbol{\beta_f}$ is the vector of parameters for the population-level effects representing here the N treatment, hybrid, presence of the transgenic trait, the growth stage, and the double and triple interaction terms among them,

and $\boldsymbol{\beta}_r$ is the vector of group-level parameters described by a normal distribution with variances $\sigma^2{}_r$. The group-level parameters represent the varying-intercepts for years and blocks nested within years, and varying-slopes among N treatments, hybrids, and transgenic event in each year and block × year. For the parameters models, we used weakly-informative priors for all traits based on normal distributions for the population-level parameters, and Gamma and Student's t-distributions for the estimated variances at the group-level effect (for the varying-intercepts and – slopes, respectively).

Supplementary Table 1. Soil parameters and test values for the study sites measured at pre-planting.

Soil information	Unit	York, NE (USA)	
		2019	2020
Organic matter †	g kg^{-1}	29.0	29.7
pH (water)		6.5	6.0
Sand content	%	14.2	11.5
Silt content	%	60.9	51.7
Clay content	%	24.9	36.8
NO_3-N ‡	mg kg^{-1}	8.9	10.5
NH_4-N ‡	mg kg^{-1}	6.5	5.8
STP §	mg kg^{-1}	80.2	20.0
STK §	mg kg^{-1}	439	478

† Organic matter (loss on ignition) for the 0 to 15 cm depth
‡ Soil nitrate and ammonium (KCl extraction) for the 0 to 60 cm depth.
§ Soil test phosphorus (Mehlich-3) and soil test potassium (ammonium acetate) for the 0 to 15 cm depth.

Supplementary Table 2. Grain yield (150 g kg^{-1} moisture), grain N concentration, total plant N uptake at maturity, and post-flowering N uptake of two WT and two DP202216 field-grown maize hybrids under 0 (N0) and 225 (N225) kg N ha^{-1} conditions during 2019 and 2020.

Nitrogen	Hybrid		Grain yield		Grain N		Total N uptake		Post-flowering N uptake	
			Mg ha^{-1}		%		kg ha^{-1}		kg ha^{-1}	
			Median	Pt > 0	Median	Pt > 0	Median	Pt > 0	Median	Pt > 0
N0	PH11V8W2Z	WT	13.5 (1.19)		1.26 (0.08)		231 (53)	73%	47 (12)	
		DP202216	14.1 (0.39)		1.30 (0.09)		229 (56)		49 (12)	
	P1421	WT	12.1 (0.3)		1.24 (0.08)		234 (47)	87%	69 (12)	87%
		DP202216	12.8 (0.66)	66%	1.26 (0.08)		220 (51)		49 (12)	
N225	PH11V8W2Z	WT	17.0 (1.19)		1.29 (0.08)		244 (42)	78%	27 (13)	72%
		DP202216	16.5 (0.38)		1.33 (0.08)		236 (46)		19 (12)	
	P1421	WT	15.7 (0.3)		1.25 (0.08)		272 (38)		58 (12)	
		DP202216	15.2 (0.66)		1.24 (0.08)		267 (42)		60 (13)	

Pt = probability for differences between DP202216 transgenic and WT control plants at each N by hybrid combination.

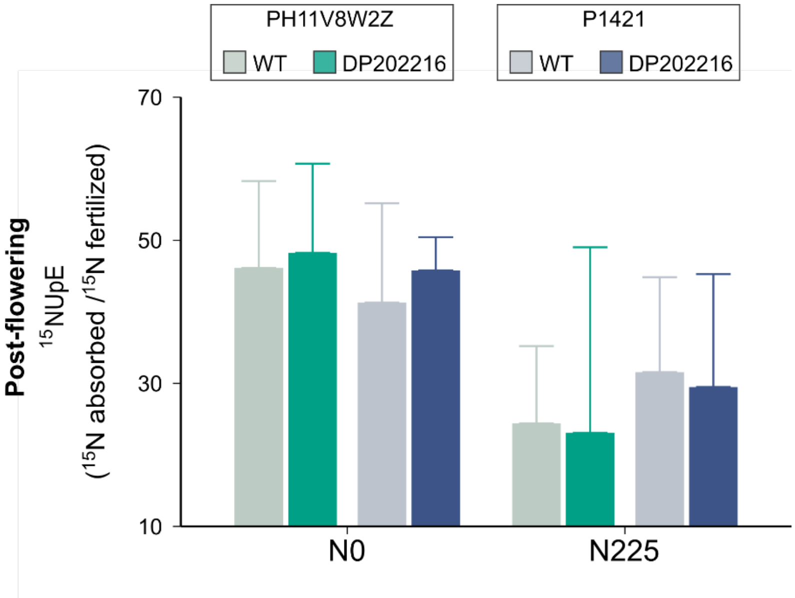

Supplementary Figure 1. ^{15}N fertilizer uptake efficiency during post-flowering stages (R1, R3 and R6) expressed in percentage over the total ^{15}N applied of two WT and two DP202216 field-grown maize hybrids under 0 and 225 kg N ha^{-1} conditions during 2019 and 2020. Bars and whiskers represent the medians and standard deviations of the posterior predictive distribution obtained by Bayesian estimation on the two-year data.

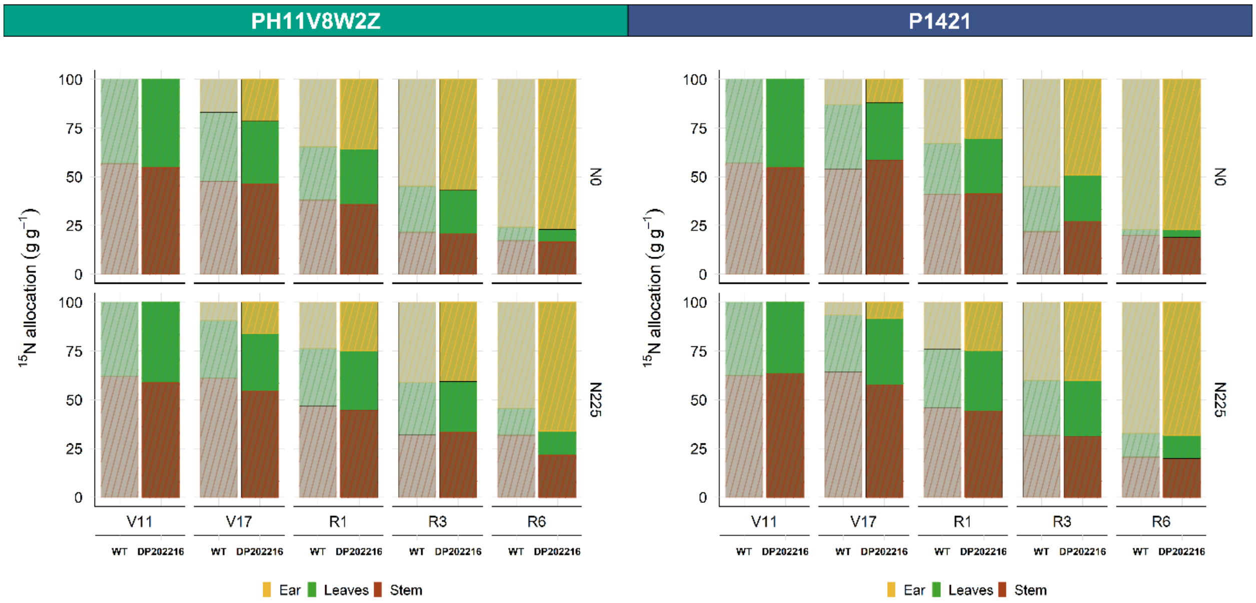

Supplementary Figure 2. Allocation of ^{15}N absorbed to green leaves, stem, and ears across maize developmental stages, expressed in percentage over the total ^{15}N absorbed, of two WT and two DP202216 field-grown maize hybrids under 0 (N0) and 225 (N225) kg N ha^{-1} conditions. Bars represent the medians of the posterior predictive distributions.

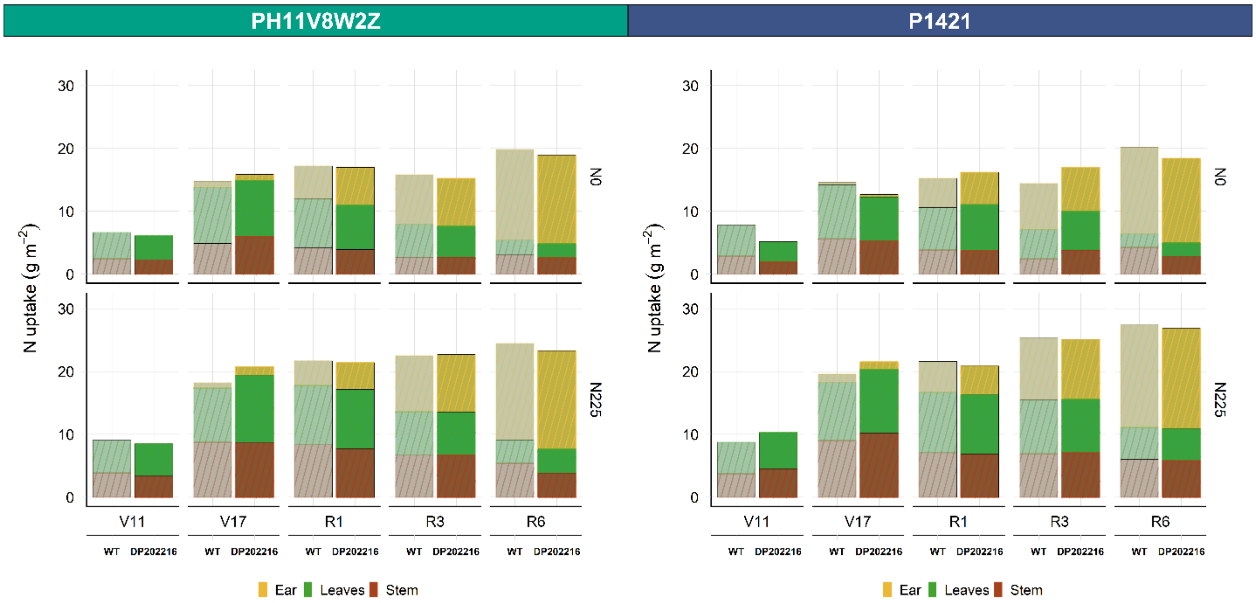

Supplementary Figure 3. Nitrogen uptake in green leaves, stem, and ears across maize developmental stages of two WT and two DP202216 field-grown maize hybrids under 0 (N0) and 225 (N225) kg N ha^{-1} conditions. Bars represent the medians of the posterior predictive distributions.

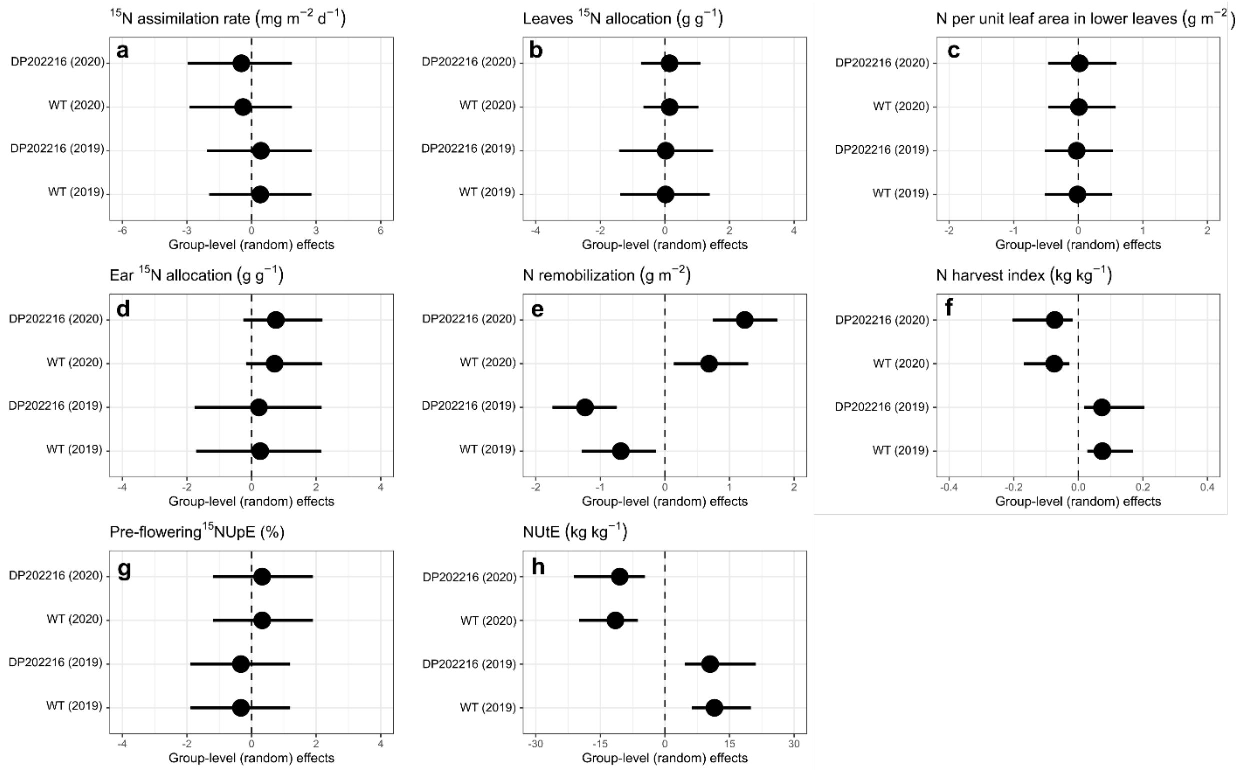

Supplementary Figure 4. Summary of distributions for group-level (random) effects coefficients for 2019 and 2020 sites across WT and DP202216 treatments in the fitted Bayesian models. Symbols and whiskers represent the medians and 95% credibility intervals of the posterior predictive distribution.

Supplementary References

1. RStudio Team. RStudio: Integrated Development for R. RStudio, Inc. Boston, MA. (2016).
2. Rosner, B. Percentage points for a generalized ESD many-outlier procedure. *Technometrics* **25**, 165–172 (1983).
3. Bürkner, P. C. brms: An R package for Bayesian multilevel models using Stan. *J. Stat. Softw.* (2017) doi:10.18637/jss.v080.i01.
4. Bürkner, P. C. Advanced Bayesian multilevel modeling with the R package brms. *R J.* (2018) doi:10.32614/rj-2018-017.
5. Douma, J. C. & Weedon, J. T. Analysing continuous proportions in ecology and evolution: A practical introduction to beta and Dirichlet regression. *Methods Ecol. Evol.* **10**, 1412–1430 (2019).
